# Philadelphia Hospital

**Published:** 1839-03-02

**Authors:** 


### CLINICAL LECTURES.

### PHILADELPHIA HOSPITAL.

*Saturday, February 16th.*—Dr. HORNER remarked:

GENTLEMEN,—In the first case which I shall introduce to day, you will recognise the woman with torpid nervous deafness, the permeability of whose Eustachian tube I tested at our last meeting with the air-douche. This operation you may remember was followed by emphysema of the pharynx, and of the face in the region of the parotid gland. As the patient had previously suffered from paralysis, and I was somewhat afraid that spasm of the glottis might occur, I ordered her to be bled to six or eight ounces. This was immediately done; no bad consequences followed the emphysema, which is now well, after making the throat uneasy for two or three days. We have since then tried the effect of the introduction of etherous vapour into the tympanal cavity. As yet there has been no amelioration, but sufficient time has not elapsed to test fully the power of this remedy. Should the treatment according to Mr. Kramer's plan, be diligently pursued for a month, I have no doubt that she will be benefited. I shall next introduce a coloured man, affected with phymosis, upon whom I propose to operate. Dr. Gibson, in the early part of the course treated so fully of this subject, that I shall not now detain you with any special account of the disease. There are, as you are aware, two modes of operating—by excision—and by slitting up the prepuce. Where the prepuce is very long the former is to be preferred. By simply slitting it open, we curtail the period of cure as well as the size of the cicatrix. I shall in the case before you operate in this way. I shall pass a director between the prepuce and the glans, and introducing a curved sharp pointed bistoury, plunge the point through the base of the prepuce and cut forwards. By this simple line of incision, the operation is secured from any interruption on the part of the patient, because should he recoil, the knife will cut the faster; moreover, a regular edge is obtained for the two laminæ of the prepuce, which should be fixed after the incision, by a stitch. [Operation done accordingly.] By cutting from before backwards, the operation is exposed to disturbance from the patient, and the risk of an irregular edge is thereby encountered. Another mode of operating recommended by Mr. Colles,* consists in making a slit near the side of the frænum. There are cases in which this is no doubt a preferable operation.

Before I dismiss this patient it will be as well for you to examine him, as he presents a remarkably good specimen of *Cataract* in the right eye. The opacity which you see on looking at his eye, might be confounded on a superficial examination with opacity of the cornea. On closer inspection, it will, however, be found posterior to the iris. He is enabled to see somewhat indistinctly at times; this takes place when the luminous rays strike the side of the cornea, and penetrate to the retina by this way. The pupil is enormously dilated, as much so as if it were produced by stramonium or belladonna. The left eye is as you see perfectly good. The rule of conduct among surgeons in such cases, is not as yet definitely settled, with respect to an operation. The general practice where one eye only is involved, is to let it alone. Now were we to operate, the focal distance in the two eyes would be different; the right eye would, therefore, as the weakest, be thrown out of the natural axis of vision. Besides, from the very lively sympathy which exists between the eyes, there is a possibility that the sound eye might become inflamed, and serious inconvenience result. If, unfortunately, any accident should happen to the left eye, then it would be proper to perform an operation, but so long as the vision of one eye remains unimpaired, nothing would be gained by it, and injury might result.

The next patient I show you, is one whom, from appearances, I should pronounce to have *Fistula lachrymalis.* I have not yet tested its existence; this I propose to do now before you. That little tumour which you see near the internal canthus of the eye, approximates the situation of such a one as attends fistula lachrymalis; but it is somewhat unusually advanced on the side of the root of the nose; also the patient is under the influence of secondary syphilis. This man, evidently, has a watery eye. He can give, in other respects, no very accurate or satisfactory account of his disease. Whenever you see that weeping at the inner canthus, accompanied with a discharge of serum and pus, you may doubt the permeability of the lachrymal passages. I will now inject tepid water through the lower punctum lachrymale. You see, no water flows from the nose, but it returns by the upper punctum and the tumour. This case is thus satisfactorily made out as fistula lachrymalis. I shall operate upon it at our next meeting. Before I send him out, I will show you his arm, which, you see, is much swollen, and read to you a history of his case, as one of syphilis.

[This was done.]

I shall, gentlemen, conclude this lecture with some remarks on *Frost-bite* or *Congelation.* The sensation of cold is relative, and depends on the degree of temperature to which we are accustomed, and the state of the body previous to its application. The media through which heat is abstracted from the body also exercise an influence, as well as the constitutional energy of the individual exposed to its loss, and the condition he may be in at the time, whether in health

* Practical Observations on Veneral Disease, p. 92, Dublin, 1837.

or disease. The phenomena of cold as shown in the human body, are very various, and are modified by the constitutional peculiarities and the age of the individual. Plethoric persons of robust frame, resist the effects of cold much better than those of a feeble, weakly habit. The extremes of youth and age, suffer also very much. The annual mortality of the children at the Hopital des Enfans Trouvès in Paris is enormous.

Local congelation is most apt to take place in parts furthest removed from the centre of the circulation. Dry cold acts less powerfully than humid, which has superadded to the direct agent cold air, the additional abstraction of heat by evaporation. According to Baron Larrey, whose opportunities of witnessing the influence of cold during the retreat of the French army from Moscow, were very great, the bad effects of cold are generally manifested on the occurrence of a thaw.

The first effect of the application of cold is a paleness of the surface, with diminution of volume, corrugation, and a disagreeable pricking sensation. The next effect is redness, with a slight tumefaction, and then a bluish tint, with pungent pain in the part. The texture now becomes soft, flexible, and shining, and assumes a slate colour, owing to the arrest of blood in it, feeling to the touch much colder than it really is, owing to its impaired vitality. In severe cases, complete insensibility and incapability of motion attend it. Where there is a very low temperature, as in northern climates, when its action is assisted by a high wind, instead of all these preparatory admonitions, the part exposed becomes suddenly marbled and insensible, or changes its colour to a dull, waxy whiteness, and becomes congealed, before the individual is apprised of what has happened. Many soldiers in the French army, during the disastrous retreat from Russia, suffered in this way. A frozen state is not absolutely a dead one, for after parts have become stiff and insensible for some hours, their vitality may be re-established. Parts which have remained for several days in a state of complete insensibility and want of myotility, have been eventually restored to their functions without being attacked with gangrene. Hence it may be inferred that the destructive influence of cold is less the result of itself than of the subsequent changes or reaction. When heat is suddenly applied, an intolerable tingling and prickling are manifested; the part tumefies, a sharp, biting heat is developed, and the pain is so intense sometimes, as to extort cries of distress. In slight cases, these phenomena disappear, but in the severer ones, the reaction being more aggravated, a vesication of the epidermis in plyctenæ follows, the spots being surrounded by a red, erysipelatous circle. In the latter respect, the analogy between a burn and a congelation is so great, that, by mere inspection, you would not be enabled to distinguish between them. The skin under these phlyctenæ becomes livid, insensible, and discoloured. In the worst cases, the vesications do not form, but there is an immediate appearance of grayish brown or black scars, which end in the sphacelation of all the part affected. In the treatment of a person who has been laboring under the influence of cold for any time, you must be particularly careful not to bring him suddenly into a heated apartment. He must first be rubbed with snow, or water mixed with pounded ice, and all your efforts must be directed to a *gradual* restoration of warmth.

Old persons, women, children, and individuals of a lymphatic temperament, are very liable to chilblains or pernio. This affection, commonly known as frost bite, is characterized by a circumscribed elevated red spot of the integuments, attended with a disagreeable heat, and a very inconvenient itching, and it runs, sometimes, into ulceration. The best applications in such cases are the terebinthinates. Some persons have recommended emollient applications, but these almost always aggravate the disease.

*Cases of Congelation from Cold.*

CASE I.—George Blubeley, aged thirty-two, entered the surgical ward January 4th, 1839; is an Englishman; nine years in America; works at weaving. On new year's day he walked about the city all the afternoon; it had commenced thawing in the middle of the day, so as to render the gutters deep; he accidentally slipped into one, thereby wetting his feet; was not intoxicated—never drinks to excess; he continued abroad until 10 o'clock; felt no sensation of chill in his feet until after sunset, when the weather became extremely cold. Upon arriving at home, his feet were very much benumbed and stiff, and his shoes and stockings were frozen fast; he was obliged to *thaw* them before they could be removed. His feet now looked a little red, but nearly natural; slept well that night. On morning of January 2d, they presented a bruised appearance, somewhat swollen. He now applied an Indian meal poultice; thought it afforded no relief, and substituted oil of turpentine; this blistered his feet, which now felt hot and cold alternately. Upon his entrance, January 4th, he had some stimulating liniment. Next day, the unguent. stramonii was substituted; at this time the nails remained, with no appearance of sphacelus; this treatment was continued until the 7th, when ulceration, with a disposition to sloughing being evident, the ungt. plumb. acet. was added to the stramonium, and continued during the day—poultices were applied at night.

CASE II.—Thomas Lester, aged fifty, entered surgical ward, January 8th, 1839. Born in England; in America nine years; is a weaver by trade; habits moderate; general health good. Two weeks before entrance, returned from England; landed at New York, and came to Philadelphia in the rail-road cars; the weather was clear, but cold. He walked about the city for several days, in his usual health. On the night of Sunday, December 29th, he felt severe pain in his right foot, which prevented his sleeping; it became very red and swollen, extending up the leg. Next day soaked his feet in bran water, without relief; the limb continuing painful, he procured some hartshorn, laudanum, and oil, and rubbed the part. After its application, the pain left the limb, but continued in the foot, which, at this time, had a dark bluish appearance, and was much puffed up. Upon his entrance, January 8th, the same appearance remained, and there was evident fluctuation of the tumefied portion, which was greatest over the metatarsal bones; he was ordered catap. ferment., with good diet, &c. This was continued until the night of the third day after, when the tumour broke, followed by a profuse discharge of bloody serum. The poultices were still applied; the skin surrounding the aperture sloughed, leaving an ulcer of tolerably healthy appearance, about three by four inches in size; this healed rapidly under the poultice—the exuberant granulations being kept down by a solution of sulph. cupri.

Case III.—John Underwood, (black,) aged 29; entered Jan. 29, 1839; born in Philadelphia; works *along shore*, as a stevedore; habits moderate. On Wednesday, Jan. 23d, a few days after the severe storm, was at work unloading a brig—worked six hours among ice and snow—before stopping work felt pain in hands extending to elbows—also in feet which were benumbed—slept none that night—hands and feet becoming swollen, he applied "scraped potatoes and beef's gall"—was somewhat easier afterwards He remained in this state until Jan. 29th, when he entered the Hospital. At the time of entrance his limbs were still swollen, with considerable increase of temperature—but the skin remained unbroken—they were then wrapped in raw cotton, which as he says "drew some and increased the heat;" it was kept on for several days—the symptoms gradually abated. Four days after entrance his feet were well—and now, Feb. 7th, all uneasy sensations have abated, except in two fingers of left hand, which are still somewhat stiff and swollen, with a peculiar hardness to the touch; there is also a dark hue beneath the nails; other changes in color not being apparent, from patient's complexion.

Case IV.—Benjamin Fisher, (black,) aged 23 years; entered Jan. 21st, 1839; born in Pennsylvania; drinks a good deal, at times getting intoxicated. Two weeks before entrance engaged carrying grain from the wharf into a store; worked all day during a snow storm; his shoes 'leaked' so that his feet were soon wet; at night his feet were painful and swollen; he could still however walk about—did nothing for them. Two weeks after he hurt his left knee, which had been formerly strained by a fall from a sleigh; on this account he entered the Hospital. At entrance his feet were still painful; they were wrapped in raw cotton, and in two days pain had subsided, and they now continue well. Patient remains on account of his knee.

---

Dr. Carswell succeeds Dr. Elliotson in the University College Hospital.

